# In pursuit of best practice through contextually relevant, accountable and responsive research

**DOI:** 10.4102/sajcd.v69i1.945

**Published:** 2022-12-12

**Authors:** Anita Edwards, Faheema Mahomed-Asmail, Anna-Mari Olivier, Jeannie van der Linde, Katijah Khoza-Shangase, Nomfundo Moroe, Joanne Neille

**Affiliations:** 1 Department of Social Science, Africa Health Research Institute, Mtubatuba, South Africa; 2 Department of Audiology, Faculty of Health Sciences, University of KwaZulu-Natal, Durban, South Africa; 3 Department of Speech-Language Pathology and Audiology, Faculty of Humanities, University of Pretoria, Pretoria, South Africa; 4 Private practice, Mossel Bay, South Africa; 5 Department of Audiology, Faculty of Humanities, University of the Witwatersrand, Johannesburg, South Africa

In this year of recovery from the coronavirus disease 2019 (COVID-19) pandemic, the authors and editors of the *South African Journal of Communication Disorders* (*SAJCD*) have, once again, contributed towards the body of knowledge in our two professions of Speech-Language Pathology and Audiology. The year 2022 has offered professionals from these professions two valuable journal issues. Issue 1 included 17 articles aimed at improving knowledge on pertinent issues such as screening and provision of services. The authors followed various methodologies, which included original research papers, an opinion paper and 3 clinical perspective pieces. Issue 2 illustrates just how busy the researchers and practitioners in our professions were during the pandemic, with 22 articles that document the challenges as well as the valuable lessons learnt during the COVID-19 pandemic regarding research, training as well as clinical service provision.

## Volume 69, Issue No. 1 (2022)

Issue 1 adds to the body of knowledge in the areas of prevention of hearing loss and language developmental delay through screening and early identification, and the provision of these services. These include ototoxicity monitoring in oncology and in community-based drug-resistant tuberculosis as well as reporting on how the lack of such programmes impacts on people’s lives. Articles on new-born and infant hearing screening programmes in primary health care facilities also expanded on existing knowledge of the prevention of hearing loss.

The opinion paper on dysphagia triage in an emergency unit and the review of evidence on the influence of screen time on language development synthesises our knowledge on these topics. The focus of the *SAJCD* extends to issues of training of future professionals addressed in the evaluation of the Community Service Programme as well as the evaluation of inter-professional collaboration for language development, and the experience of health professionals when treating patients with communication and or swallowing disorders.

## Volume 69, Issue No. 2 (2022)

Issue 2 was dedicated to exploring the impact of COVID-19 on Speech Language and Hearing (SLH) professions in low-middle-income countries (LMICs), with a direct focus on COVID-19’s impact on research, teaching and clinical service provision. Topics reported on fell into three main themes. The first theme evaluated the impact of the pandemic on conducting clinical research in SLH, a key component of ensuring best practice in both training and clinical care provision.

The second theme was that of clinical training of future SLH practitioners during the pandemic with the constraints resulting from the pandemic. The topics that were reported on included the use of simulations for clinical training, using intensive writing programmes to develop critical thinking, online teaching and learning and the use of tele-supervision during clinical training.

The third theme reported on the impact of the pandemic on our service provision. This included providing treatment for aphasia, neonatal feeding, speech, language and swallowing services and audiological rehabilitation, where the environments of residential care homes and hospitals were the contexts for some of the studies. In this theme, the impact of the virus on middle ear status and the cochlea-vestibular system was also explored.

The real impact of these research initiatives and publications will however only be of value if we ensure that we do not stop at simply disseminating the knowledge in peer-reviewed journals, but that we move into finding practical ways to use the knowledge and evidence provided to inform best practice in our clinical settings. We need to apply these findings to actively change how we provide services to our clients and how we prepare the next generation of practitioners as well as how we prepare for the next global pandemic. Knowledge translation into practice will close the gap between the knowledge published in our journal and our practice.

The key words for knowledge translation are ‘use’ and ‘action’. Some potential actions for researchers, practitioners and professional associations as well as academic institutions are to actively use the knowledge shared on these issues as suggested by the authors. For example, ‘the use of a dysphagia triage checklist could have implications for patient health outcomes’. Other practical examples are ‘multidisciplinary teamwork’ and ‘simplified national ototoxicity monitoring protocol’. Further recommended actions and uses focus on ‘urgent Community Service Programme review, with the aim of standardising and redefining its intended outcomes and pertinent criteria’, and some mentioning ‘careful and systematic planning’ and ‘strategies to increase resources’.

## Our mandate

In some countries, and for some research funders, knowledge translation from research into practice is embedded in the mandate. The call to action by all SLH professionals is essential to prevent the knowledge generated from lying dormant on the journal pages. It is also incumbent on us to be collaborating inter-professionally to ensure widespread improvement of services to our clients, as was done in the research where collaboration with colleagues in engineering was achieved around the use of artificial intelligence and machine learning in our professions. Another example was exploring communication difficulties by healthcare workers when interacting with patients with communication disorders.

Advocacy for the use of knowledge both by our clients and practitioners and importantly by policy and decision makers is the responsibility of all SLH professionals, not only the authors and editors of journals. As the SAJCD’s 2022 Editor-in-Chief, Section Editors and Guest Editors, we urge all authors and practitioners to take our mandate seriously and ensure the relevance of our research on our training, research and clinical service provision.

